# Comparison of Resorption in Autogenous Dorsal Onlay Cartilage Grafts: An Experimental Study

**DOI:** 10.1007/s00266-024-04134-7

**Published:** 2024-05-28

**Authors:** Fatih Öner, Günay Kozan

**Affiliations:** 1 Department of Otorhinolaryngology, Faculty of Medicine, Kastamonu University, 37150 Kastamonu, Turkey; 2 Department of Otorhinolaryngology, Faculty of Medicine, Dicle University, Diyarbakır, Turkey

**Keywords:** Autogenous cartilage, Cartilage resorption, Rhinoplasty, Rabbit model

## Abstract

**Objectives:**

The present study was designed to compare the graft resorption characteristics of autogenous cartilage from the septum, auricle, and costal in the superficial muscular aponeurotic system of the nasal dorsum of the rabbit model.

**Methods:**

Equal-sized perichondrium-free septal, auricular, and costal cartilage grafts were collected from fifteen New Zealand white rabbits. Cartilage grafts were taken at the scale of two grafts from each animal’s ear, two from its costal part, and one from its septum. Costal cartilage grafts that were shaped with a micro-motor device and monopolar electrocautery, elastic cartilage grafts that were shaped with a micro-motor device and monopolar electrocautery, and septal cartilage grafts that were shaped with a scalpel were all implanted into the dorsum of rabbit’s noses to create five groups. All autogenous cartilage tissues were removed 3 months later. Cartilages were evaluated for histological features, graft mass, and chondrocyte density resorption.

**Results:**

The elastic cartilage group, where electrocautery was used to shape the cartilage, had a higher resorption score than the other groups. The costal cartilage graft shaped with a micro-motor was also observed to have the best cartilage regeneration score.

**Conclusion:**

We observed that the resorption of costal cartilage was lower than that of ear and septum cartilage. It was determined that micro-motor application for the shaping process caused less resorption and stimulated more regeneration than cautery application.

**Level of Evidence III:**

This journal requires that authors assign a level of evidence to each article. For a full description of these Evidence-Based Medicine ratings, please refer to the Table of Contents or the online Instructions to Authors www.springer.com/00266.

## Introduction

Cartilage grafts are used in almost all areas of otorhinolaryngology practice. Although they are primarily used in rhinoplasty of the nose for functional and esthetic purposes because they consist mainly of cartilage structures, cartilage grafts are used to reconstruct areas structurally composed of cartilage or places that require support.

Several types of grafts are widely used in rhinoplasty, including autografts, allografts, and heterografts [1]. The ideal source for all kinds of grafts in rhinoplasty is autogenous cartilage grafts, and they have been used in esthetic and reconstructive surgery for craniofacial and nasal defects in the last few decades. Cartilage tissue is ideal for grafting because it can perform anaerobic glycolysis, survives low oxygen levels, and is well tolerated by the host. [2]. Autologous grafts, obtained from different locations in the body and used for nasal reconstruction, are primarily composed of bone and cartilage. The cartilage tissues can be obtained from the nasal cartilage and the ear to reconstruct minor defects, and the costal cartilage can reconstruct major nasal structural defects [3].

Consensus regarding use of different materials has yet to be established in clinical practice. Studies have measured the resorption levels of cartilage grafts, which are widely and successfully used in clinical settings, both qualitatively and quantitatively [4]. Also, the cartilage grafts (ear, costal, or rib) are more likely to be resorbed depending on the preparation and type of the graft [5, 6].

The rabbit model has been used for decades as a human surrogate to examine various histopathologies encountered by the reconstructive surgeon [3, 7]. We also used the New Zealand White Rabbit to compare the resorption levels in autogenous cartilage grafts obtained from different parts of the body and the effect of shaping by monopolar electrocautery and micro-motor on the resorption and viability of implanted cartilage tissues.

The present study evaluated the resorption level of different autogenous cartilages taken from the septum, auricle, and rib. In addition, we used other techniques, such as scalpel, electrocautery, and micro-motor, in shaping the grafts to compare graft resorption under the superficial musculo-aponeurotic system (SMAS) of the nasal dorsum of rabbits.

## Materials and Methods

### Animal Housing

Fifteen male New Zealand rabbits (36 weeks old), weighing 2200–2500 g, were used in the study. The experimental procedures were performed under the protocol approved by the Ethical Committee (for Experimental Animal Care and Use) of the Faculty of Veterinary Sciences at Bingöl University (Decision No: 2020/03-03/02). The study was conducted at the Experimental Animal Studies Laboratory of the Bingöl University Experimental Research Center. We selected the rabbit model in the study since rabbit cartilage tissue is similar to human cartilage tissue and is an established recipient site model for nasal dorsal implants [8]. The rabbits were fed complete rabbit chow pellets and kept individually in small cages with a solid or meshed wire bottom at 20–24 °C.

### Surgical Procedure

**Fig. 1:**
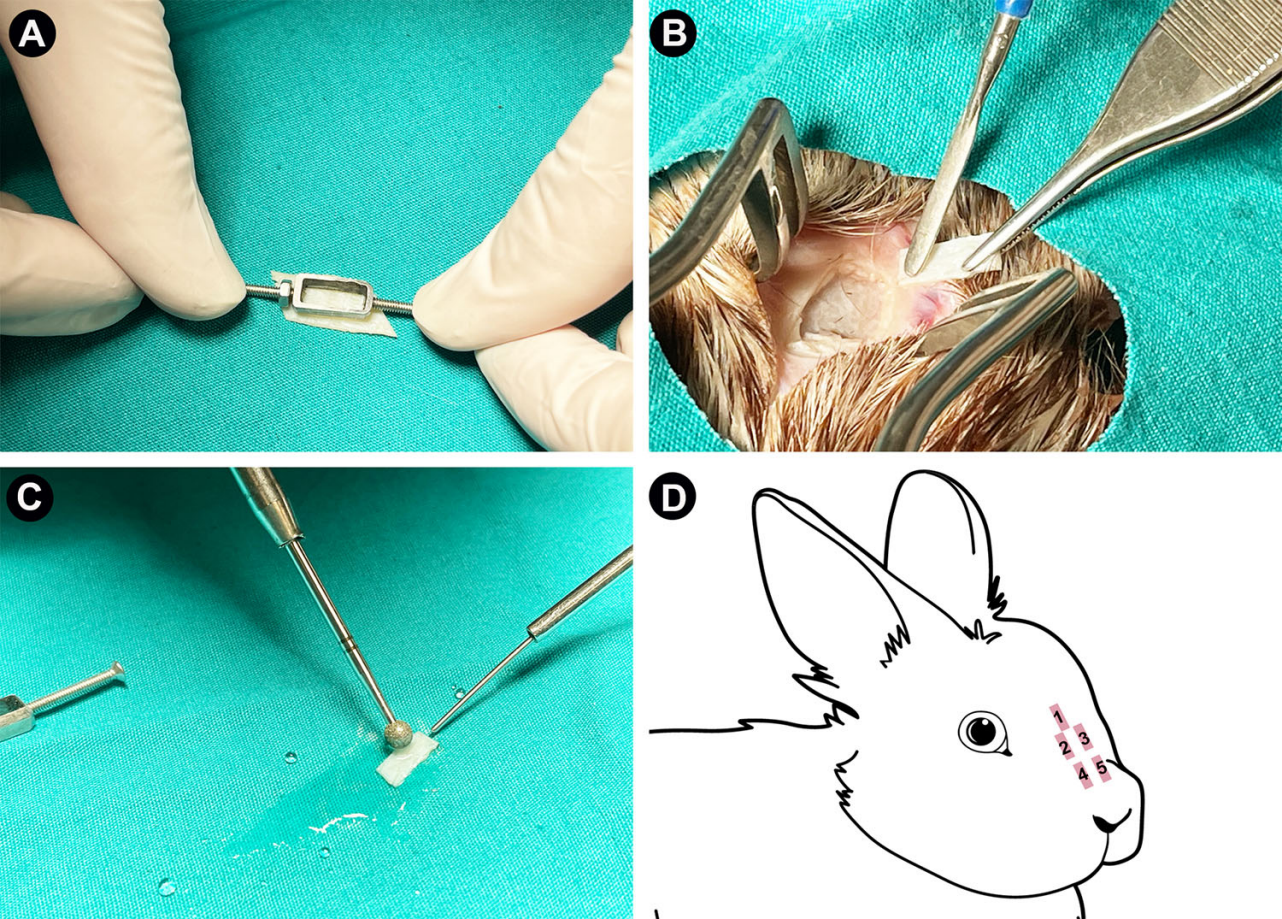
Cartilage preparation stages for the groups: **A** all cartilage grafts were diced as 10 × 5 × 1 mm sizes, **B** one of each ear and costal grafts were reshaped with the use of monopolar electrocautery, **C** one of each ear and costal grafts were reshaped with the use of micro-motor devices, **D** all cartilage grafts were implanted in the nasal dorsum of the rabbits

Before the operation, the rabbits were anesthetized with xylazine (Rompun, 10 mg/kg i.m.) and ketamine (Ketasol, 100 mg/kg i.m.), and then, costal, elastic (from the ear), and septum cartilages were removed from each rabbit. The nasal septum was reached from the caudal part, and the septal cartilage was dissected and removed from the mucous membranes. The perichondrium was removed from all cartilage grafts with a surgical blade. Before putting cartilage specimens into the body, all of the cartilage that was collected was reshaped with scalpel, monopolar electrocautery or micro-motor devices (Primado 2, NSK, Tochigi, Japan) to make them all the same size (10 × 5 × 1 mm). Only a scalpel was used in the septal cartilage group to shape the graft. We corrected the cartilage grafts overflowing from our standard mold in monopolar electrocautery spray mode, utilizing the coagulation button and 20 W energy until shaving reduced them to 1 mm thick. All graft specimens were weighed with a precision balance device, disinfected with a 70% alcohol solution, and implanted under the SMAS of the nasal dorsum of each rabbit. In the implantation procedure, two costal and two auricular cartilages and one septal cartilage graft were placed in the SMAS of the nasal dorsum of the rabbits. The incision areas were sutured with an absorbable 3-0 Vicryl suture. Also, the ear and other operation areas were sutured with Vicryl and cleaned using povidone-iodine. The preparation of cartilage harvesting stages is presented in Fig. 1. Animals were divided into five groups, as below:

- Group 1: Costal cartilage graft shaped by drilling with a micro-motor device
- Group 2: A monopolar electrocautery device-shaped costal cartilage graft
- Group 3: Drilling with a micro-motor device to shape elastic cartilage grafts
- Group 4: A monopolar electrocautery-shaped elastic cartilage graft
- Group 5: Septal cartilage graft shaped by a scalpel

All operations and the preparation of graft implantations were performed in 2 days. After the procedure, a 10 mg/kg dose of ceftiofur (Excede, Zoetis) was subcutaneously injected once a week for 4 weeks. No infection was observed in the operation areas during the postoperative period. Three months after the operation, all rabbits were euthanized with an intracardiac lidocaine injection (Jetokain amp, 3 ml), and all grafts were removed and freed from surrounding tissues. The facial mesostructure was removed en bloc for histological study. After the facial mesostructures of the animals were carefully dissected, the implanted grafts were removed. At the end of the experiment, five cartilages from fifteen rabbits (45 in total) were removed from the dorsum of the nose, and their weights were weighed with a precision scale. Then, three samples were randomly selected from each cartilage (215 samples in total), cartilage thickness and histological properties were evaluated, and resorption degrees were compared.

### Histological Analysis

For collecting cartilage under anesthesia, all cartilage tissues were removed and fixed in a 10% neutral buffered formaldehyde solution. Then, these tissues were dehydrated and embedded in paraffin, and serial sections were cut using a microtome (Leica RM2125RT, Leica Microsystems, Wetzlar, Germany). Afterward, we obtained nine serial and three randomly selected slides for each animal, which were used for histochemical staining.

### Crossmon Modified Mallory’s Triple Staining

Randomly selected, three sections of each animal were deparaffinized and rehydrated. Three slides were stained with Crossmon modified Mallory’s triple protocol for histopathologic evaluation and the determination of collagen density [9].

### Verhoeff–Van Gieson Staining

Randomly selected, three sections of each animal were deparaffinized and rehydrated. Three slides used in the histological evaluation of elastic fibrils were stained with the Verhoeff–Van Gieson staining protocol [10].

### Toluidine Blue Staining

Randomly selected, three sections of each animal were deparaffinized and rehydrated. Three slides for histological evaluation of proteoglycans and glycosaminoglycans were stained with the toluidine blue staining protocol [11].

### Histological Evaluation of Cartilage Tissues

For the histological evaluation of cartilage tissues, a modified Colombo score was used to assess the amount of possible change [12]. These changes are loss of superficial layer, erosion, fibrillation, cyst, osteophyte, loss of proteoglycan, disorganization of chondrocytes, clonal chondrocytes, exposure of subchondral bone, and subchondral vascularization. The scores were as follows:

- 0: No change was observed
- 1: The feature was observed but was weak
- 2: The feature was pronounced and well defined

All histological evaluations were analyzed blindly, and the scores were determined by averaging the values found by evaluating three different sections of the cartilage tissue belonging to each rabbit. Then, the average group score was calculated by summing these scores. To evaluate cartilage tissues in terms of resorption, the cartilage tissues were stained with three different histochemical staining methods.

The numbers of nucleated lacunae and the basophilic staining nucleated lacunae were counted, respectively, to evaluate the regeneration capacity of cartilages. Afterward, according to a previous study, we calculated the basophilic staining nucleated lacunae ratio and recorded it as a percentage [3]. Each cartilage tissue graft was scored as follows:

- (0) = No nucleated lacunae were observed
- (+ 1) = 1–25% Nucleated lacunae
- (+ 2) = 26–50% Nucleated lacunae
- (+ 3) = 51–75% Nucleated lacunae
- (+ 4**) **= More than 75% nucleated lacunae observed in the specimen

### Histological Measurement of Cartilage Thickness

**Fig. 2:**
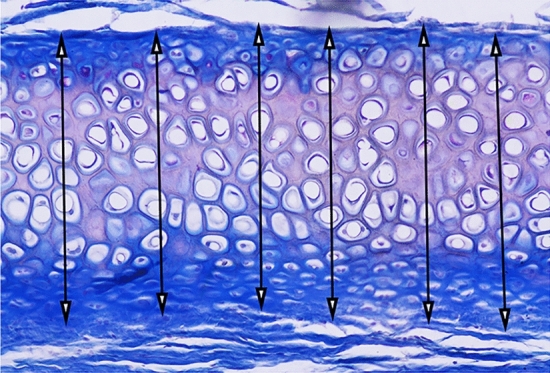
Histological measurement of cartilage tissues thickness. Arrows: measurement distance of cartilages

Cartilage thickness was used as an indicator for calculating the resorption of cartilage. Assessments were conducted by a blinded researcher utilizing commercially accessible imaging software (ImageJ program). Measurements of cartilage thickness were obtained from multiple sections (comprising one midline and three each on the left and right sides), as illustrated in Fig. 2. These individual measurements were then aggregated to compute the mean resorption values for each respective section. Subsequently, the mean resorption values of the sections were averaged to obtain a mean sample thickness.

### Statistical Analysis

The graft weight values were given as mean ± SD for the group’s mean values. The Wilcoxon test was used to compare the values obtained before and after the operation. All analyses were performed using SPSS software (version 20 for SPSS, Chicago, IL, USA), and *p *< 0.05 was considered significant.

## Results

### Evaluation of the Weight of Cartilage Before and After Operation

**Fig. 3:**
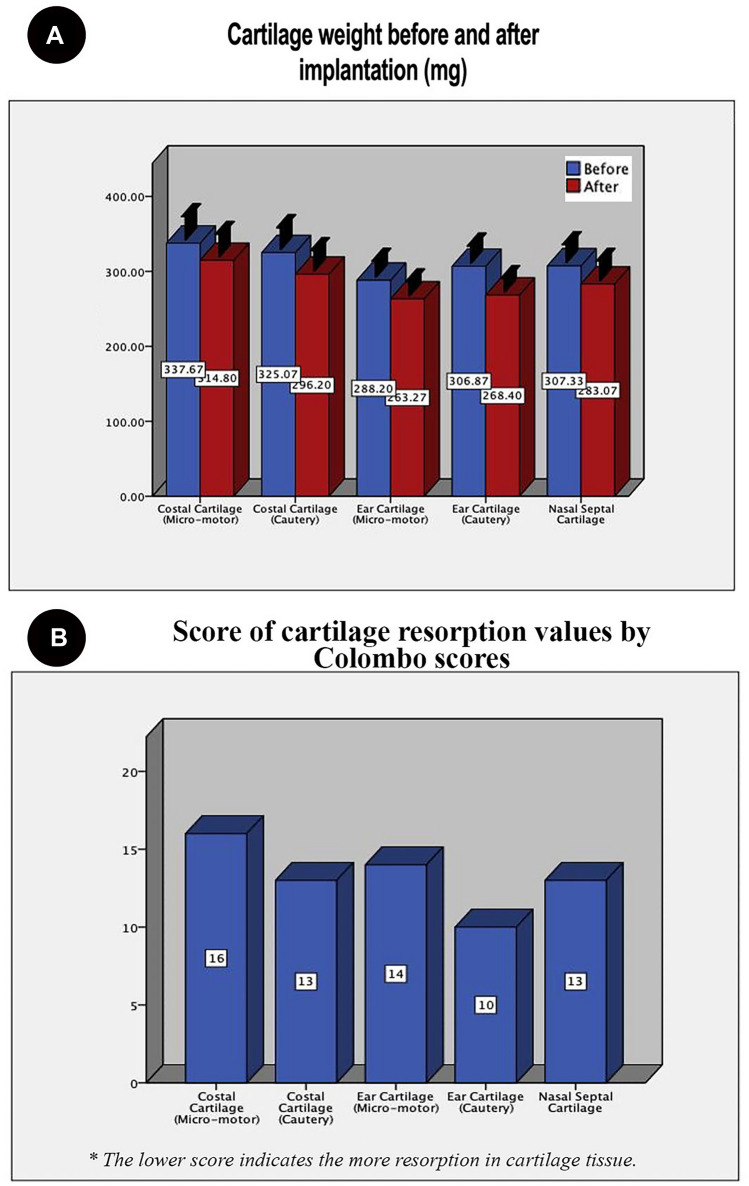
Graphs of cartilage grafts evaluations. **A** Cartilage grafts weight before and after implantation of SMAS, **B** score values of cartilage resorption by Colombo scores

In the evaluation of the weight loss of cartilage grafts, it was found that the mean weight after implantation was significantly lower than before implantation (*p *< 0.001). The highest weight loss (resorption) was determined in the ear cartilage shaped with a monopolar electrocautery group regarding the difference in pre- and postoperative weights of cartilages. In addition, there was a significant difference among the study groups in terms of weight change percentage (*p *< 0.001). The averages and percentage changes in weight measurements before and after the implantation are presented in Table 1 and Fig. 3.

**Table 1:** Weight of the grafts before and after the operation of implantation of SMAS

Groups	Before	After	Change		*P*
			mg	%	
Costal cartilage (micro-motor) group 1	337.7 ± 32.2	314.8 ± 31.5	22.9 ± 6.6	6.8 ± 1.9	0.001
Costal cartilage (cautery) group 2	325 ± 30.3	296.2 ± 28.3	28.9 ± 5 .5	8.9 ± 1.6	0.001
Elastic cartilage (micro-motor) group 3	288.2 ± 22.4	263.3 ± 19.7	24.9 ± 8.7	8.6 ± 2.8	0.001
Elastic cartilage (cautery) group 4	306.9 ± 23.9	267.8 ± 19.2	38.5 ± 11.8	12.4 ± 3.3	0.001
Septal cartilage (scalpel) group 5	307.3 ± 25.6	283.1 ± 27.3	24.3 ± 4.98	8 ± 1.9	0.001

The values expressed as mean ± standard deviation. The paired samples *t *test was used to compare the before and after groups’ difference values. *P *< 0.05 was considered significant

### Histological Results

**Fig. 4:**
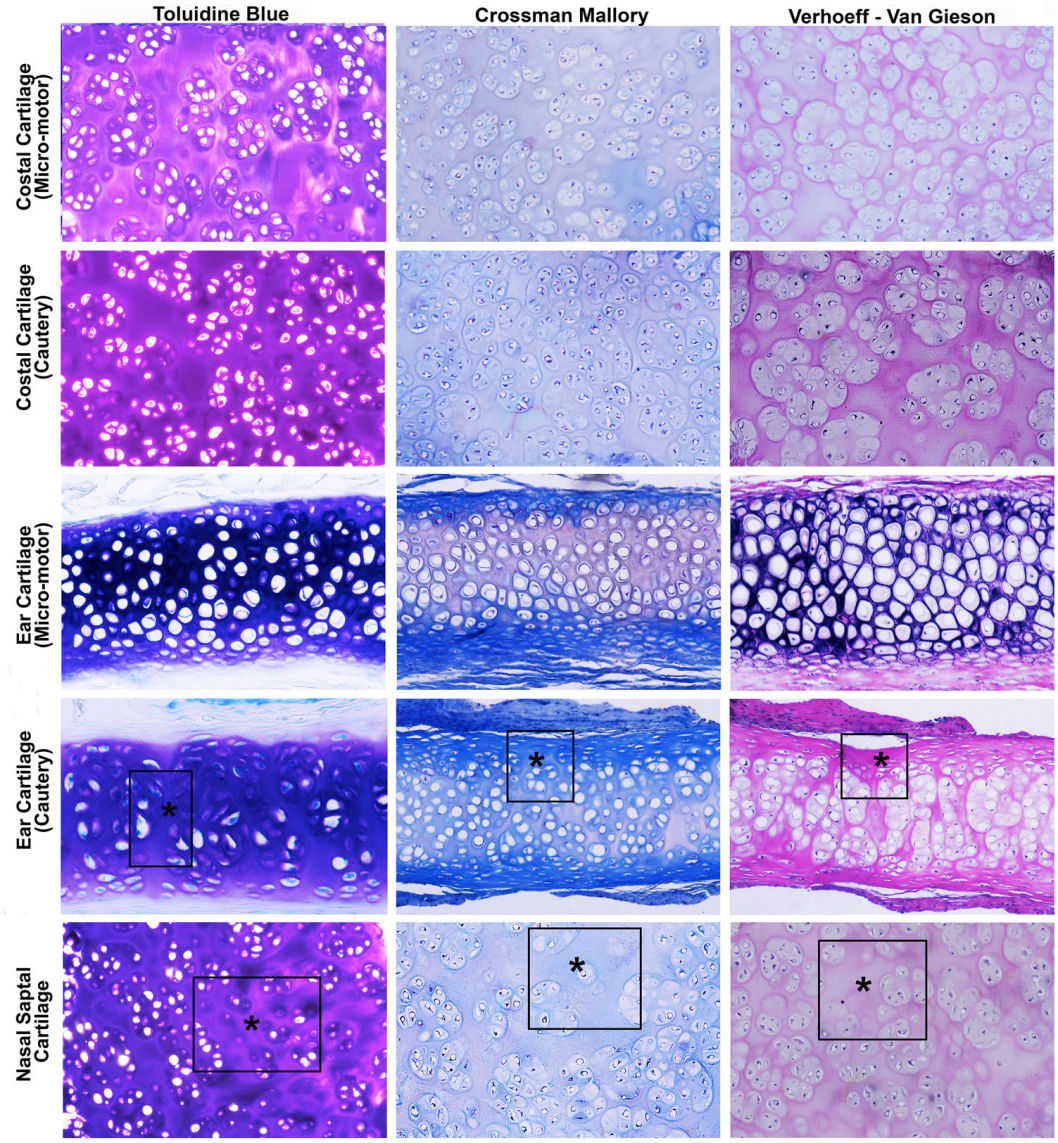
Illustration of histological sections of the cartilage tissues staining with toluidine blue, Crossmon modified Mallory, and Verhoeff–Van Gieson; square and asterisks indicate the resorbative and decreased amount of matrix as well as chondrocyte-like cells

The cartilage resorption scores of Group 1 were higher than those of the other groups. Additionally, the scores of costal grafts shaped with the surgical micro-motor group, the elastic cartilage graft shaped with a micro-motor, and the septal cartilage graft shaped by a scalpel were higher than the scores of the other groups. The lowest score for resorption of cartilage graft was determined in the ear cartilage shaped with a monopolar electrocautery group. The resorption scores of all graft specimens are presented in Table 2 and are shown in Figs. 3 and 4.

**Table 2:** Evaluation of cartilage resorption values in terms of Colombo scores

Parameters	Costal C.		Elastic C.		Septum C.
	Micro-motor (group 1)	Cautery (group 2)	Micro-motor (group 3)	Cautery (group 4)	Scalpel (group 5)
Cell morphology	2	2	2	2	2
Matrix staining	2	2	1	2	1
Structural integrity	2	1	2	1	2
Thickness/defect filling	1	1	2	1	2
Osteochondral junction	0	0	0	0	0
Basal integration	2	2	1	1	2
Cellularity	2	2	2	1	2
Clustering/distribution	2	2	2	2	2
Adjacent cartilage degeneration	1	1	1	0	0
Non-inflammation	2	1	1	0	0
Total scores	16	13	14	10	13

C., cartilage

The scores followed as 0 = these changes were not observed; 1 = the feature was observed, but was weak; and 2 = the feature was pronounced and well defined. The lower score indicates the more resorption in cartilage tissue. The high score favors normal cartilage tissue

In evaluating the regeneration capacity of cartilages, it was found that the score of nucleated lacunae with basophilic staining was 2 (above 50% nucleated lacunae) in the ear cartilage shaped with monopolar electrocautery group, 3 (60–70% nucleated lacunae) in the costal cartilage graft shaped with monopolar electrocautery group, elastic cartilage graft shaped with a micro-motor group, and septal cartilage graft shaped by a scalpel, and 4 (above 75–80% nucleated lacunae) in the costal cartilage graft shaped with a micro-motor group (Fig. 4).

**Fig. 5:**
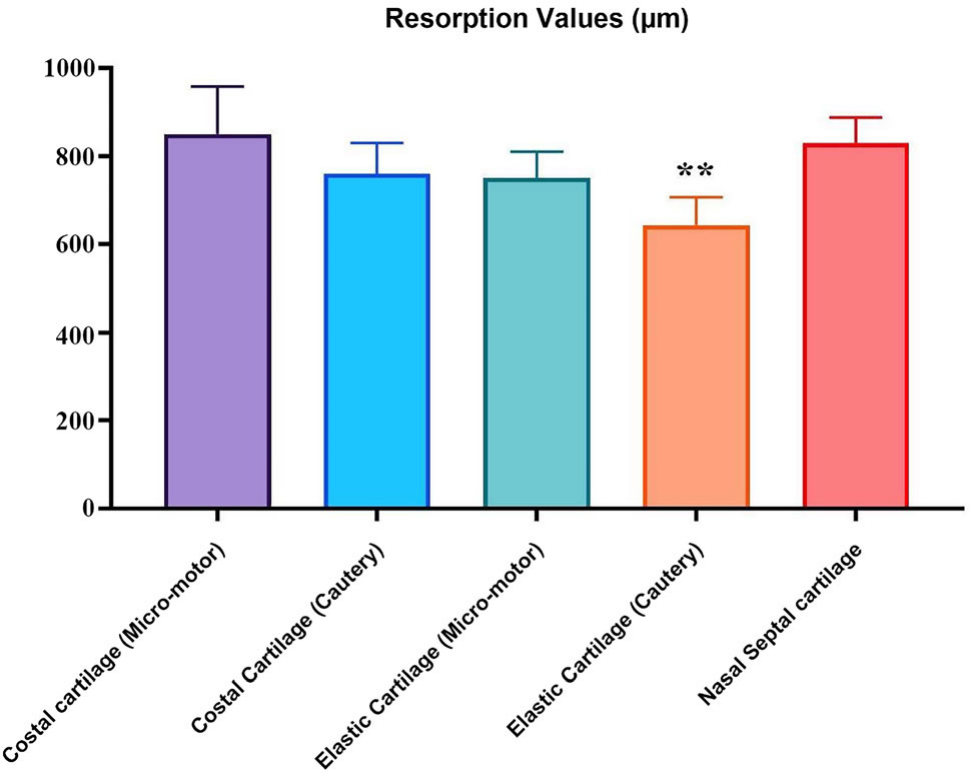
Graphical representation of cartilage thickness. The group with the greatest decrease in thickness was the elastic cartilage electrocautery group

The results of cartilage thickness measurements are shown in Fig. 5. Specifically, the thickness of cartilage was observed to be greater on the costal cartilage (micro-motor) in comparison to other types of cartilage. Conversely, the lowest thickness was noted in the elastic cartilage (cautery). Using Pearson correlation analysis, the relationship between 5 different groups was examined. No statistically significant difference was found between the groups for cartilage thickness, which we considered as another indicator of cartilage resorption. By taking three samples from each cartilage (a total of 225 samples), cartilage thicknesses were measured in microns and compared (Table 3).

**Table 3:** Comparison of cartilage thicknesses between groups, average thickness values, and their correlation with each other

	Thickness ± SD (micrometers)	*n*	Costal cartilage (micro-motor)	Costal cartilage (cautery)	Elastic cartilage (micro-motor)	Elastic cartilage (cautery)	Nasal septal cartilage (scalpel)
Costal cartilage (micro-motor)	903.53 ± 48.1	45	1	0.165*	0.91*	0.842*	0.951*
Costal cartilage (cautery)	810.07 ± 43.2	45	0.165*	1	0.848*	0.908*	0.604*
Elastic cartilage (micro-motor)	794.22 ± 60.3	45	0.91*	0.848*	1	0.931*	0.444*
Elastic cartilage (cautery)	654.67 ± 62.5	45	0.842*	0.908*	0.931*	1	0.162*
Nasal septal cartilage (scalpel)	771.78 ± 64.1	45	0.951*	0.604*	0.444*	0.162*	1

Pearson correlation test (*P *< 0.05), P*

## Discussion

In this study, we try to find the resorption of the different cartilage types in the nasal dorsum of an experimental rabbit model. We evaluated the weight loss and histological resorption degrees of standard-sized and shaped costal, auricular, and septal cartilages that we placed on the nasal dorsum of rabbits and compared the results.

Correction of the nasal dorsum is one of the critical stages of rhinoplasty surgery. The operations performed on the back of the nose are conducted for esthetic purposes rather than functional purposes. The irregularities on the dorsum after removal of the nasal hump with the use of a chisel or rasp are usually corrected with the help of well-processed autogenous dorsal onlay cartilage grafts. Costal and auricular cartilages are commonly used as autogenous graft sources, mostly in revision cases. Autogenous costal cartilage is the most widely used material in reconstructing nasal structures [13]. Irregularities may occur in the nasal dorsum depending on the degree of resorption that develops in the cartilage over time after surgery. In addition, the amount of resorption may also vary depending on how the cartilage is processed with the tools used during the surgery. Dorsal onlay grafts are placed on the cartilage and bone skeleton under the skin, helping to shape the skin and irregular dorsum. Some researchers have used soft autogenous tissue grafts such as temporal muscle fascia or dermal grafts [14]. Temporal muscle fascia is a tissue resistant to resorption but cannot provide sufficient volume alone since it is thin [15].

Autogenous cartilage grafts such as conchal, septal, and costal cartilage are often preferred in facial reconstructive and cosmetic surgeries. Conchal and septal cartilage would generally be insufficient for correcting nasal deformities such as a flat nose. Nonetheless, some irregularities also occur on the nasal dorsum, depending on the materials’ resorption over time [16]. Although cartilage grafts allow for shaping to a certain level, they return to their original state due to their structure’s physical and biomechanical characteristics, leading to delayed postoperative deformities [17]. In the present study, it was aimed to determine whether there was a difference between the resorption rates of autogenous costal, auricular, and septum cartilage grafts obtained from rabbits and the resorption rates (micro-motor and electrocautery) depending on the physical applications used to shape the auricular and costal cartilage grafts.

It was shown in many studies that cartilage grafts can easily take the shape of the area where they are applied due to their flexibility; thus, they are frequently used in reconstructive surgery [3]. It has been reported in some studies that both crushed and non-crushed cartilage grafts can be used successfully between 85.5 and 93.5% in nasal esthetic operations [18]. However, one of the most critical issues related to cartilage grafts in the nose is the resorption that the graft can undergo and the degree of reshaping [19]. Our results indicated that in pre- and postoperative weight measurements of cartilage materials, costal cartilage graft application was found to be less resorbed, and its regeneration score was higher. It was observed that the highest resorption and the lowest regeneration scores were in the auricular cartilage.

It has been reported in human studies that irradiated costal cartilage homografts have been extensively studied, and their resorption levels have varied between 0 and 75% [20]. On the other hand, in animal studies, the resorption rates of irradiated cartilage homografts implanted in the sheep facial skeleton have been found to vary between 1 and 20% [21]. A study on cats revealed that fresh cat costal autografts and homografts were minimally resorbed, while frozen and fresh autografts and irradiated homografts were significantly resorbed [22]. In a study conducted by Tjelmeland and Stal to compare the resorption of rabbit auricular and costal cartilage, it was determined that the auricular cartilage placed on the rabbit nasal dorsum was resorbed at a rate of 18.5%, and those implanted in the control region (occiput) at a rate of 14.5%. However, they observed no significant resorption in the costal cartilage [19].

The study determined the better score values in the costal that maintained their vitality. Chondrocytes do not undergo any degenerative changes, and the hyaline matrix has no invasion and/or resorption. Lattyak et al. [8] reported lower resorption in the costal cartilage than in the septal and auricular cartilage. Adlington et al. [23] determined that the resorption of the crushed mouse costal cartilage homografts (irradiation, formalin, glutaraldehyde, and alcohol) placed on the rabbit dorsum was higher than that of uncrushed rabbit cartilage homografts. The results of our study have indicated that the electrocautery and micro-motor procedures applied to auricular and costal cartilage autogenous grafts affected resorption. In addition, less resorption has been detected in the cartilage grafts shaped with the micro-motor. We think that the reason why absorption is less in those using micro-motors, unlike other methods, is that the motor increases tissue density by slightly pressing on the tissues with pressure during rotation. Although there is structural resorption in the placed cartilage grafts, it should be considered that the healing tissues formed by the surrounding tissues can physically compensate for this loss. One of the limitations of our study is that the tissues (wound healing, granulation, and fibrosis) formed around the cartilage grafts were not shown ultrasonically, which reduces our chance of making further comments on this situation.

As a limitation of the study, we can say that the change in the thickness of the tissues around the cartilages was not measured ultrasonically after the cartilages were placed. For new studies, we suggest placing cartilages in different parts of experimental animals and comparing the resorption. Again, whether cartilage resorption will be higher when the experimental period is kept longer may be the subject of new studies.

In conclusion, the costal cartilage had less resorption than the auricular and septum cartilage. Electrocautery, when utilized as a solution to trim the excess parts of the cartilage, causes more cartilage resorption than other procedures. We recommend that it be taken into consideration that the resorption occurring in the grafts may cause deformity at the point where they are placed in the postoperative period.

## Abbreviation

SMAS: Superficial musculo-aponeurotic system
